# Investigation on status and needs of pharmacy intravenous admixture service (PIVAS) personnel training: A qualitative focus group interview study

**DOI:** 10.1097/MD.0000000000036309

**Published:** 2023-12-15

**Authors:** Chunsong Yang, Zheng Liu, Yaya Yang, Yong Hu

**Affiliations:** a Department of Pharmacy, West China Second University Hospital, Sichuan University, Chengdu, China; b Evidence-Based Pharmacy Center, West China Second University Hospital, Sichuan University, Chengdu, China; c NMPA Key Laboratory for Technical Research on Drug Products In Vitro and In Vivo Correlation, Chengdu, China; d Key Laboratory of Birth Defects and Related Diseases of Women and Children, Sichuan University, Ministry of Education, Chengdu, China; e West China School of Medicine, Sichuan University, Chengdu, China; f Department of Pediatrics, West China Second University Hospital, Sichuan University, Chengdu, China.

**Keywords:** focus group interview, personnel training, pharmacy intravenous admixture service, qualitative study, training needs

## Abstract

This study aimed to investigate the status and the needs of personnel training in pharmacy intravenous admixture service (PIVAS) and provide a reference for the comprehensive training of pharmacists in PIVAS in China. A total of 21 pharmacists from PIVAS of West China Hospital, Sichuan University and West China Second University Hospital, Sichuan University were selected by purposive sampling to conduct 3 rounds of focus group interviews, and the Colaizzi phenomenological method was used for qualitative analysis. The personnel training in PIVAS is mainly on practical skills for daily work but rarely on comprehensive quality, adopting centralized lectures and regular assessment while lacking in individualized training. The needs of staff focus on career development, which was settled through improving comprehensive ability in training, especially scientific research ability, for solving problems in daily work and obtaining corresponding outputs. PIVAS personnel training should pay more attention to comprehensive quality and scientific research ability, and establish an individualized, refined, and specialized training system to meet the needs of talent development and improve the quality of pharmacy services.

## 1. Introduction

Pharmacy intravenous admixture service (PIVAS) is an emerging division of the pharmaceutical department in medical institutions in recent years. It is a place where the intravenous drugs of the whole hospital are centralized admixture under a clean environment.^[1]^ For ensuring the efficiency of PIVAS and the quality of finished infusion, the staff must have sufficient expertise and professional ability. The guidelines for the construction and management of PIVAS, issued by the National Health Commission of the People Republic of China in December 2021, explicitly required that the professional pharmacy technicians engaged in centralized admixture of intravenous drugs should receive pre-job professional knowledge training, pass the assessment, and regularly accept continuing education in pharmacy.^[2]^

PIVAS personnel training is the core work to ensure the safety, economy, and effectiveness of the clinical intravenous medication. A previous systematic review including 5 literature related to the PIVAS personnel training in China showed that quite a difference existed in training content, training mode, training objectives, evaluation indicators, etc. among different hospitals.^[3]^ In addition, according to a multicenter cross-sectional survey, the scientific research training of PIVAS staff in Chinese hospitals is insufficient and the corresponding output is relatively low, with influencing factors including regions, professional titles, education background, and employment natures.^[4]^ However, the current published studies were only reflecting the mode and needs of current PIVAS personnel training in China through quantitative data, and lacking a direct understanding of the preferences and attitudes of those who indeed received training.

The Focus group interview is one method of qualitative study, which organize 6 to 12 homogeneous participants to discuss a topic for 2 hours under the guidance of one moderator according to the predetermined process and tasks in a relatively open, transparent, and stress-free environment.^[5]^ It is an effective tool to assist researchers to discover and explore problems through collecting a large amount of valuable qualitative data in a short discussion process, which complements the deficiency of quantitative study in understanding the needs, attitudes, and perspectives of participants at a deeper level.^[6,7]^

To make up for the shortcomings of previous studies, this qualitative study aimed to conduct focus group interviews to evaluate the existing training content and mode, and further explore the training needs of PIVAS staff, providing a reference for establishing comprehensive and process-oriented personnel training.

## 2. Methods

### 2.1. Study design

The pharmacists of PIVAS from West China Hospital, Sichuan University and West China Second University Hospital, Sichuan University were purposive sampling to capture a variety of stakeholder perspectives for 3 rounds of focus group interviews from May to June 2021. The research team consisted of 3 members, one associate chief pharmacist (team leader, as well as PIVAS leader), one pharmacist-in-charge and one pharmacist (team members, as well as PIVAS staff), who had all participated in qualitative research training, and were responsible for theme selection, interview conduction, and data analysis in this study.

### 2.2. Participants and settings

The inclusion criteria of participants in the focus group interview were (i) those currently working in PIVAS; (ii) good at communication and strong in expression; and (iii) willing to participate in this study. We approached potentially eligible staff volunteering to participate in this study, of which were finally included in consideration of the strategic orientations based on their gender, age, education background, and work experience for increasing their representativeness. All participants write an informed consent to participate and had the right to withdraw at any time without giving any reason. Based on the principle of information power, we determined the sample size sufficient to answer our research question.^[8]^

The first round of interviews was started with PIVAS staff from West China Second University Hospital, Sichuan University at the PIVAS office, and the second and third rounds were respectively interviewed with the staff from West China Hospital, Sichuan University at their office.

### 2.3. Theme

The research team reviewed the literature and initially developed interview themes. The themes were finalized after pre-interviews and revisions under the guidance of qualitative research experts.

Theme 1: Status of PIVAS personnel training, including training content and training mode;

Theme 2: Needs of PIVAS personnel training, including career development and scientific research.

### 2.4. Focus group interview and data collection

The research team and the interviewees arrived at the site at the agreed time. The team leader conducted the interview process, and 2 members respectively recorded the facial expressions, tone of voice, pauses, and body language of the speakers during the interview, without other unrelated persons at the interview site. Before formally starting the interview, the interviewees read and signed the informed consent form and then filled out the self-made interviewer demographic information form. The moderator gave an opening speech, introduced the purpose and method of the interview concerning the themes, clarified the rules of discussion and encouraged the interviewees to actively communicate with each other, and then guided the interviewees with open-ended questions to express their cognition according to the predetermined process and tasks for 2 hours. During the interview, the interviewers also asked some probing questions based on the answers. After the interview completion and no new information emergence, the moderator briefly summarized and thanked the interviewees before ending the interview.

### 2.5. Data analysis

The research team coded and categorized data within 24 hours after each round of interviews, with 2 members working back-to-back to integrate the audio recordings and interview notes into textual materials. Disagreements were resolved by discussion among 2 analysts or by third-party adjudication. The Colaizzi descriptive phenomenological method^[9]^ was used to analyze the interview data: (i) carefully read all textual materials; (ii) extracted content relevant to this study; (iii) summarized, distilled, and coded meaningful content; (iv) organized and classified the coded content and then formed themes; (v) related the themes to the interviewees and narrated them in detail; (vi) stated the essential structure generating the phenomenon; and (vii) returned the collated information to each interviewee for verification and ensuring the quality of the study.

### 2.6. Quality assurance

To reduce the bias of results, a series of measures for quality assurance^[10]^ were applied during the study: (i) the interview moderator was a PIVAS leader who was experienced in management; (ii) the moderator did not express personal opinions and would encourage interviewees to freely express their attitudes and actively communicate with each other for collecting more valuable perspectives during the focus group interviews; (iii) the members recorded the scenes in detail when interviewees spoke, and combined with the voice information to understand their true thoughts to the greatest extent; and (vi) the collated information was returned to each interviewee for verification to ensure that the preferences and attitudes of individuals were accurately reflected in our study.^[9]^

### 2.7. Ethical considerations

This study has been approved by the Ethics Committee of West China Second University Hospital, Sichuan University. Written informed consents were obtained from participants before the interviews.

## 3. Results

### 3.1. Characteristics of participants

In total, 21 PIVAS staff received the interview, including 17 females and 4 males, with a mean age of 28 years old (range: 23–37 years old) and a mean work experience in PIVAS of 3.8 years (range: 1–6 years). The interviewees consisted of 2 pharmacists-in-charge, 14 pharmacists, and 5 assistant pharmacists, with the degree of 2 masters, 11 bachelors, and 8 colleges. All interviewees were coded as N1-N21 in check-in order (Table 1).

**Table 1:** Characteristics of participants in focus group

	No.	Gender	Age	Degree	Professional title	Work experience in PIVAS (yr)
Round 1	N1	Female	25	College	Assistant Pharmacist	3
	N2	Female	24	College	Assistant Pharmacist	1
	N3	Female	27	Bachelor	Pharmacist	4
	N4	Male	29	Bachelor	Pharmacist	5
	N5	Female	35	Master	Pharmacist-in-charge	6
	N6	Female	27	Bachelor	Pharmacist	3
	N7	Female	31	Bachelor	Pharmacist	5
Round 2	N8	Male	30	Bachelor	Pharmacist	4
	N9	Female	26	Bachelor	Pharmacist	3
	N10	Female	23	College	Assistant Pharmacist	1
	N11	Female	24	College	Assistant Pharmacist	1
	N12	Male	28	College	Pharmacist	5
	N13	Female	31	Bachelor	Pharmacist	4
	N14	Female	27	Bachelor	Pharmacist	3
Round 3	N15	Female	29	College	Pharmacist	4
	N16	Female	37	Bachelor	Pharmacist-in-charge	7
	N17	Female	30	Master	Pharmacist	2
	N18	Male	26	College	Pharmacist	4
	N19	Female	25	Bachelor	Pharmacist	2
	N20	Female	25	College	Assistant Pharmacist	3
	N21	Female	28	Bachelor	Pharmacist	4

### 3.2. Theme 1: Status of PIVAS personnel training

#### 3.2.1. Training content.

The training content of PIVAS staff in West China Hospital, Sichuan University and West China Second University Hospital, Sichuan University mainly focuses on practical skills in daily work, continuously improved through a series of ways such as pre-job training and daily handover meeting, etc. The staff of each position share their work experience and learn from each other, thus enhancing the collective level of PIVAS staff.

“When we first entered the PIVAS, we had to attend pre-job training to know what PIVAS did, learn various related systems, and accumulate professional knowledge. Most importantly, we should remember the responsibilities and the operating procedure of each position that we must be acquainted with in the future, as well as some emergency plans for hospital infections, water, and power outages, etc. There are a lot of things needed to be learned after getting down to work, especially hands-on skills like the operation of finished infusions.” (N3)

“Our department has a handover meeting at noon every day, and the colleagues on duty often talk about the matters that happened in their work and we discuss the solutions. Meanwhile, the prescription reviewers introduce the special situations encountered and provide a reference for learning and strengthening professional knowledge. The dispensers share the mistakes as warnings for avoiding similar mistakes in the future. And they will also pose the problem of consumables preparation, constantly optimizing the method of supplementing consumables.” (N7)

The interviewees pointed out that PIVAS has less comprehensive quality training, including problem-solving ability, communication ability, collaboration ability, scientific research ability, psychological quality, etc., which is mainly improved through daily accumulation and self-summary.

“The daily workload of PIVAS is so large and the training is mainly related to the practical work, such as common errors or problems and newly discovered admixture skills, but relatively less to the problem-solving ability, communication ability, and teamwork ability, which was built up on our own in daily work.” (N12)

“After working in PIVAS, I have learned that some problems encountered in daily work are needed for searching the literature, but I’m not familiar with databases or academic websites, even sometimes unclearly know where to search, which I think is one of the scientific research abilities needed to train. In addition, When I first came to work, I felt huge pressure and anxiety about what I couldn’t understand or finish by myself. I heard that some psychological quality training was carried out in other hospitals, which is appropriate and nice to provide some humanistic care and psychological counseling.” (N19)

#### 3.2.2. Training mode.

The training of PIVAS staff mainly adopts centralized lectures based on daily work needs, and regular assessment according to professional titles and working experiences.

“Our department has weekly business learning for training. The reporter of the centralized lecture often introduces the indications and dosage from drug instructions and guidelines in detail, involving off-label use as well. There will be corresponding exams after the centralized lectures. The usual personnel training is mainly carried out in the form of meetings, for example, the dosage and operation of a new drug will be explained during the handover meeting at noon. If there have new equipment introduced, we will also call for learning together, so that we can be familiar with its use as soon as possible. Besides, the staff has regular and corresponding continuous assessments according to their titles and working experiences.” (N4)

For new employees, trainees, and interns, PIVAS adopts the model of centralized lectures combined with one-on-one teaching, but the training effect is not ideal due to the rapid turnover of positions, which is a disadvantage to individualized training.

“The new employees, trainees, and interns firstly get to know what PIVAS does in general and what they mainly do, then they will enter the corresponding positions according to the schedule and be taught by the experienced staff who work in the position on that day. However, there is a problem with such one-on-one teaching. Different staff on the duty may lead to different operations and other skills. Thus we plan to make a standardized teaching template so that the students master it as soon as possible. But the students rotate relatively quickly now who switch to other departments when they just get familiar with the last one, adverse to individual, fining and professional training.” (N16)

### 3.3. Theme 2: Needs of PIVAS personnel training

#### 3.3.1. Needs of career development.

The PIVAS staff were busy with their daily work and had no time to consider their career development, looking forward to participating in training about career development.

“The risk and load of working in PIVAS are high and large. I just want to finish my work quickly every day, and only think about how can I promote my professional title on the regular assessment of our department every year. As for what else I can do in the future, or to what extent I can overcome to improve my position, I don’t have any time for careful consideration because of the huge workload. Therefore, I hope I could have the opportunity to attend some training related to career development for the future, at least to understand how I should set the career development goals and implementation plans.” (N13)

Due to the lack of sufficient scientific research ability, professional ability and communication ability, some PIVAS staff have certain difficulties in career development. Therefore, they hope to comprehensively improve their ability by taking part in corresponding training or going out to study and communicate.

“The hospital requires scientific research outputs and article publication for the professional title promotion. But it is difficult for me to publish articles since I graduated from college. I hope that there will be training in scientific research, writing skills, and other aspects in the future. Besides, I think we should communicate more with the clinic and learn more about medication to accumulate professional ability. I also want to improve my abilities in various aspects, such as responsibility, initiative, and discipline.” (N18)

“In fact, we hope to have more opportunities to exchange to better hospitals to study and discover the different working ways among us, drawing lessons to improve our work efficiency. We can also participate in more academic conferences, learn some good experiences from home and abroad, optimize the workflow, and improve our ability in all aspects.” (N21)

#### 3.3.2. Needs of scientific research.

In China, scientific research output is often used as one of the indicators for a professional title promotion. The corresponding output of PIVAS staff is relatively low due to the lack of systematic scientific research training. The interviewees expected that PIVAS can strengthen the training on scientific research ability, solve the problems in daily work through scientific research methods and obtain certain scientific research outputs to meet the needs of career development.

“PIVAS is a good place to perform research, but the output of PIVAS is relatively low because we don’t have systematic training in scientific research and a complete research team in our division. We hope that PIVAS can organize relevant systematic training regularly on topic selection, literature search, study design, statistical analysis, writing ability, etc., to stimulate interest and raise awareness increasingly. Based on these abilities, the problems we encountered in the future can be solved with the available evidence.” (N5)

“From my perspective, I hope to carry out more training on topic selection, scientific research thinking, and innovation, for exploring valuable and meaningful research directions in the work, publishing high-quality articles and making achievements in scientific research, further expanding work area and improving career value.” (N17)

## 4. Discussion

This study collected qualitative data based on focus group interviews, which is currently less applied to the research about PIVAS personnel training, and we found that the current training of PIVAS staff is mainly on practical skills in West China Hospital, Sichuan University and West China Second University Hospital, Sichuan University. The purpose of continuous training on practical skills for daily work is to familiarize staff with and continuously strengthen the knowledge and skills required for their work, and keep in mind the obligations, risks, and legal responsibilities assumed in the work, further providing safe, quality and effective of finished infusions for patients. Meanwhile, the major mode of PIVAS training is centralized lectures and regular comprehensive assessments, as new employees, trainees, and interns are trained to combine with one-on-one teaching. This way with the addition of heavy workloads had occupied the most time of PIVAS staff, accordingly reducing the comprehensive quality training in problem-solving ability, communication ability, psychological quality, etc., and had inadequate time and energy to participate in individualized training or career development training.

A multicenter cross-sectional survey on the current status of PIVAS personnel training in 137 medical institutions in China showed that the main contents of training for PIVAS staff included professional theoretical knowledge, practical operation abilities, pre-job training, and standard operating procedures, while the training of communication skills, teaching ability, management ability, management tools, comprehensive ability development, and career development planning is relatively small, which is consistent with the results of our study.^[11]^ Moreover, the training modes are lectures and practical operations, mainly 1–2 times a month, and less than half of the staff were satisfied with the training, indicating that the current PIVAS personnel training system still needs further improvement and optimization.

In addition, PIVAS staff expect to improve their comprehensive ability in all aspects to meet the needs of career development, which requires them to participate in various training and communication related to career development meanwhile doing their job well. Shen et al mentioned in their study that the number of career planning training was the only factor that significantly affected career planning scores,^[12]^ suggesting that we need to increase training to meet the career development needs of PIVAS staff so that they can develop a clear recognition of career development and gradually realize it in daily work.

Among 501 valid questionnaires returned from 29 hospitals reported by Yang et al,^[4]^ 72.1% of PIVAS pharmacists had a moderate and higher need for scientific research training, 82.4% had a moderate and higher interest in research, but only 16.6% of PIVAS pharmacists had published papers during their work. Based on the above quantitative study, our study further collected and deeply explored the need of PIVAS personnel training, and most of the interviewees strongly expressed strengthening the corresponding training in scientific research. Another cross-sectional study from the perspective of PIVAS leaders investigated the current status of training and research,^[13]^ and most leaders believed that PIVAS staff had a high demand for scientific research, but fewer had mastered the methods, with relatively poor ability. It may be related to the busy daily work of PIVAS leading to the lack of attention to scientific research, and the staff only give concerned with finishing the daily work, while ignoring the role of scientific research in guiding practice. The application of scientific research methods not only can effectively solve problems in daily work but also can stimulate innovative spirit, thinking, and ability, further optimizing the workflow and improving work efficiency. On the other hand, the lacking of the systematic training in scientific research may lead to the rigid thinking mode and loss of ability to solve problems independently among PIVAS staff, which further hinder the creativity and development of the department, and the adverse impact may finally reflect on patients. Therefore, by increasing the scientific research training for PIVAS staff and forming scientific research interest groups, we can stimulate interest and cultivate thinking in scientific research, motivate the staff to actively participate in scientific research, and obtain corresponding outputs for meeting the needs of career development.

Under the premise of ensuring the quality of practical skills training, we suggest that it is necessary to further strengthen the comprehensive quality and individualized training. After long-term comprehensive training, PIVAS will gather a high-quality, efficient, and professional talent team. At the same time, the staff will have sufficient time to develop their career, and good career development will motivate them to actively participate in various training and academic exchanges, obtain higher quality research outputs, and gradually build up a benign training system in PIVAS.

There are some methodological limitations in our study. Firstly, this study conducted focus group interviews with PIVAS staff only in 2 hospitals, and the coverage was small which might not reflect the actual situation in other regions with different training mode. However, the results of this qualitative study were consistent with the previous quantitative study that investigated across the nationwide, which improve the reliability of our conclusions to a certain extent. Besides, the focus groups were conducted after the working activity, and the PIVAS leader served as the moderator. Although we facilitated the meetings to enable everyone to express, the asymmetric relationships between participants and moderators were established ahead of the focus groups and may have affected the participants’ responses.

In conclusion, based on the results of this qualitative study, we suggest that in addition to the training of practical skills in daily work, the PIVAS should further strengthen comprehensive quality training, attach importance to individualized, refined, and specialized training, and pay close attention to the training needs of staff, increasing the training efforts in career development and scientific research. A comprehensive and process-oriented training mode not only facilitates the provision of high-quality pharmacy services to patients but also promotes the development of the PIVAS and continuously improves the quality of hospital services.

## Acknowledgments

We thank all the participants who took part in this study. We would also like to thank the affiliated institutions and organizations for providing methodologies and valuable comments.

## Author contributions

**Conceptualization:** Chunsong Yang.

**Data curation:** Zheng Liu, Yaya Yang.

**Formal analysis:** Yong Hu.

**Methodology:** Zheng Liu.

**Resources:** Chunsong Yang.

**Software:** Yaya Yang.

**Writing—review & editing:** Yong Hu.

**Writing—original draft:** Chunsong Yang, Yaya Yang, Yong Hu.
